# The impact of metal amino acid complexes on cuticle quality and *Salmonella* Enteritidis contamination in laying hens’ eggs

**DOI:** 10.3389/fvets.2025.1692361

**Published:** 2026-01-30

**Authors:** Saruanna M. S. Clemente, Mércia R. Barros, Carlos B. V. Rabello, Marcos Jose Batista dos Santos, Waleska R. L. Medeiros-Ventura, Rogério Ventura da Silva Junior, Felipe P. Melo, Priscila O. Silva, Fábio A. B. Santos, Raquel Burin, Alba Fireman

**Affiliations:** 1 Universidade Federal Rural de Pernambuco, Recife, Brazil; 2 Instituto Aggeu Magalhães (FIOCRUZ/PE), Recife, Brazil; 3 Zinpro Corporation, Eden Prairie, MN, United States

**Keywords:** egg production, food safety, *Salmonella* Enteritidis, eggshell quality, trace minerals

## Abstract

**Introducion:**

Eggshell quality and microbial safety are critical concerns in poultry production, with *Salmonella* Enteritidis contamination representing a significant public health risk. Traditional inorganic mineral supplementation may not optimize eggshell integrity against bacterial penetration. This study investigated the effects of different metal–amino acid complexes on eggshell cuticle quality and resistance to *S.* Enteritidis penetration in egg laying hens.

**Material and methods:**

Two experiments were conducted with 67-week-old Dekalb White laying hens with treatments consisting of inorganic minerals (IM; Control at 100% recommendations inclusion) or different trace mineral inclusion rates (100, 70, and 40%) as either amino acid-complexed minerals (AACM, Experiment 1) or lysine and glutamic acid-complexed minerals (LGCM, Experiment 2). The quality of the eggshell cuticle was measured using spectrophotometric analysis, and experimental contamination with *S.* enteritidis was performed to evaluate bacterial penetration after various storage periods.

**Results:**

Supplementation with 40% AACM improved shell thickness and palisade layer values compared to IM (*p* < 0.01). LGCM supplementation at 70 and 40% levels enhanced cuticle visual staining scoring (*p* < 0.01). Eggs from hens fed 40% AACM reduced *Salmonella* contamination, with 91.7% of samples classified as having no risk for consumption. All LGCM treatments completely prevented *S.* Enteritidis penetration into egg yolks regardless of inclusion level. In conclusion, AACM improved eggshell quality and reduced *S.* Enteritidis contamination in eggs.

**Conclusion:**

Supplementation with 40% AACM resulted in 91.7% of samples being free of yolk contamination, while LGCM supplementation at all levels completely prevented bacterial penetration into egg yolks, achieving 100% safety despite eggshell contamination.

## 1 Introduction

Eggshells are an important structure in eggs. They act as a protective barrier, defending the egg’s contents from physical damage, dehydration, and microbial contamination (1). This protective function appears central to maintaining both nutritional value and consumer safety. When eggshell quality becomes compromised, the economic consequences for the egg industry can be substantial, primarily through breakage and defects (2). Since hen age, genetics, diet, environmental conditions, and storage determine eggshell quality (3, 4), careful attention across the entire production chain is essential. The scale and urgency of this challenge are amplified by current market dynamics, with global primary egg production reaching approximately 90.6 million tons in 2022 (5). Even small improvements in eggshell quality can bring substantial economic benefits while addressing consumers’ growing emphasis on food safety, particularly regarding *Salmonella* risk mitigation. Indeed, the egg industry has developed strong commercial brands across its product portfolio in response to consumers’ demands for eggs with improved safety profiles, lower contamination risk, and transparent production practices. As a result, eggshell quality and *Salmonella* prevention are crucial brand differentiators that impact consumer purchasing decisions. In response to these converging economic and consumer demands, the industry has been exploring nutritional strategies to improve eggshell quality as a comprehensive solution addressing both profitability and food safety.

One of the major public health concerns related to egg safety is the contamination by non-typhoidal *Salmonella* serotypes, which are the leading causes of foodborne illnesses worldwide (6, 7). Globally, in 2017, it was estimated that non-typhoidal *Salmonella* invasive disease was responsible for approximately 535,000 illnesses, 77,500 deaths, and 5,680,000 disability-adjusted life-years (8). Within eggs, *Salmonella enteritidis* (*S.* Enteritidis) is the most commonly detected serovar, followed by *Salmonella* Typhimurium (9). The common practice of consuming raw or undercooked eggs heightens the risk of illness, making eggshell integrity a critical line of defense (10).

The eggshell is covered by a cuticle, a thin layer of organic material. This structure seals the pores, preventing bacteria from penetrating the eggs (11). A major weakness in commercial egg production is still inadequate cuticle deposition, which allows pathogens to contaminate its contents. Therefore, to address this challenge, the poultry industry has focused on dietary trace mineral supplementation to strengthen both the mineralized eggshell structure and support optimal cuticle formation, thereby enhancing the overall antimicrobial barrier (11).

Strategic supplementation with minerals such as manganese (Mn), zinc (Zn), and copper (Cu) has been shown to enhance eggshell thickness and breaking strength by supporting the organic matrix and improving mechanical properties (12–14). Recent research highlights the benefits of metal amino acid complexes, which demonstrate superior bioavailability compared to inorganic sources (15, 16). For example, organically bound Mn, Zn, Cu, and chromium (Cr) improve absorption and retention in hens, optimizing calcium utilization and reducing mineral excretion (17, 18).

Gao et al. (19) demonstrated that Cu complexation with lysine and glutamic acid significantly enhanced mineral bioavailability via increased absorption, suggesting these amino acids facilitate mineral transport. However, the impact of metal–amino acid complexes, including those specifically complexed to lysine or glutamic acid (LGCM) and those complexed to diverse essential amino acid ligands (AACM), on their potential to decrease *Salmonella* penetration in eggs remains unclear.

Understanding how mineral supplementation specifically enhances cuticle quality and prevents *S.* Enteritidis penetration is important for improving food safety in commercial egg production. While improvements in overall shell strength are well-documented, the precise effects of mineral form and bioavailability on the protective capacity of the cuticle are unexplored.

This study aims to investigate the impact of different mineral supplementation strategies on eggshell cuticle quality and resistance to *S.* Enteritidis penetration. It compares conventional inorganic sources of Zn, Mn, Cu, iron (Fe), selenium (Se), and iodine (I) with organic complexes either with unspecified amino acids or specifically with lysine and glutamic acid. By elucidating the impact of these different mineral strategies on cuticle quality and *S.* Enteritidis penetration. The research seeks to refine dietary practices in the egg industry, offering evidence-based strategies to reduce contamination risks and enhance egg safety for consumers.

## 2 Materials and methods

### 2.1 Animal management and ethics

The research protocol was approved by the Animal Research Ethics Committee (CEUA) of the Federal Rural University of Pernambuco (CEUA n° 95/2018). The experiment was conducted at the Experimental Station of Small Animals of Carpina (EPAC), Federal Rural University of Pernambuco, Carpina, Pernambuco, Brazil. A total of 560 Dekalb White laying hens that were 67-week-old were evenly split into two experiments, housed in conventional wire cages (five birds per cage; cage dimensions: 100 × 40 × 45 cm). Each cage was equipped with trough-type feeders and nipple drinkers.

### 2.2 Experimental design

In Experiment 1 (Exp. 1), the effects of supplementing the diet of laying hens with amino acids-complexed minerals (AACM) on eggshell cuticle quality and resistance to *S.* Enteritidis penetration were evaluated. In Experiment 2 (Exp. 2), the effects of supplementing the diets of laying hens with lysine and glutamic acid complexed minerals (LGCM) on these same response variables were assessed.

For each experiment, birds were allocated to treatments using a completely randomized design. Each experiment included four treatments with eight replicates per treatment and five birds per replicate.

The supplementation treatments for Experiment 1 were:

1. IM: 100% inorganic minerals (control)
2. AACM-100: 100% amino acid-complexed minerals
3. AACM-70: 70% amino acid-complexed minerals
4. AACM-40: 40% amino acid-complexed minerals.

The supplementation treatments for Experiment 2 were:

1. IM: 100% inorganic minerals (control)
2. LGCM-100: 100% lysine and glutamic acid complexed minerals
3. LGCM-70: 70% lysine and glutamic acid complexed minerals
4. LGCM-40: 40% lysine and glutamic acid complexed minerals.

### 2.3 Animal and management

The experimental period lasted 154 days, consisting of a 14-day adaptation period followed by five 28-day production cycles. Feed and water were provided *ad libitum* throughout the experiment. The daily lighting program consisted of 17 h of daily light (12 h of natural light and 5 h of artificial light). Environmental conditions were monitored daily using data loggers (HOBO U12-013) installed at the center of the house and digital thermohydrometers (Incoterm, model 7663.02.0.00) positioned at multiple locations across the facility. The average daily temperature and relative humidity during the experimental period were 31 °C and 68%, respectively.

### 2.4 Diets and mineral premix formulation

The experimental diets were formulated to meet or exceed the nutritional requirements specified in the Dekalb White management guide (20), except for the trace mineral concentrations that varied according to the experimental treatments (Table 1). All diets were isoenergetic and isonitrogenous, with 2,820 kcal/kg metabolizable energy and 16.4% crude protein.

**Table 1:** Composition of experimental diets.

Ingredients %	Content
Ground corn	58.8
Soybean meal	23.8
Soy oil	2.76
Calcite limestone	10.7
Meat and bone meal, 44%	1.58
Sodium bicarbonate	0.05
Salt	0.345
DL-methionine, 99%	0.234
L-lysine, 78.8%	0.14
L-threonine, 98.5%	0.068
Adsorbent^1^	0.1
Probiotic^2^	0.05
Phytase^3^	0.006
Vitamin premix^4^	0.1
Mineral premix^5^	0.285
Inert^6^	1.054
Total	100
Chemical composition (%)	
Metabolizable energy kcal kg^−1^	2,820
Dry matter^7^	90.0
Crude protein	16.4
Crude protein^7^	16.2
Ash^7^	15.1
Digestible methionine	0.450
Digestible methionine + cystine	0.680
Digestible lysine	0.860
Digestible threonine dig	0.610
Digestible tryptophan dig	0.170
Calcium	4.50
Available phosphorus	0.370
Sodium	0.180
Chlorine	0.270
Potassium	0.600
Crude fat	5.49

Supplied per kilo of product: ^1^Guarantee levels: bentonite 666 g kg^−1^; beta glucans 54 g kg^−1^; mannanoligosaccharides 59.4 g kg^−1^; bio-actives phytogenic 16.5 g kg^−1^; ^2^Guarantee levels: Bacillus licheniformis (min) > 16×1010 UFC g^−1^; ^3^Guarantee levels: phytase (min) 10,000 FTU/g; ^4^Guarantee levels: (kg kg^−1^ of product):vitamin A 8,000 IU; vitamin D3 2,000 IU; vitamin E 10,000 IU; vitamin K3 2,000 mg; vitamin B1 1,000 mg; vitamin B2 4,000 mg; vitamin B6 2,500 mg; vitamin B12 11,000 mg; niacin 25 g; calcium pantotenate 10 g; folic acid: 550 mg; and biotin: 50 mg; ^5^The composition is presented in Table 2; ^6^Sand; ^7^Values analyzed.

The mineral premixes used in each experimental diet contained Zn, Mn, Cu, Fe, Se, and I at the concentrations presented in Table 2.

**Table 2:** Trace minerals calculated and analyzed in water, diets, and experimental premixes.

Diets	Zn	Mn	Fe	Cu	Se	I
	mg/kg					
Calculated diets						
IM	60	70	40	8	0.25	1
100*	60	70	40	8	0.25	1
70*	42	49	28	5.6	0.175	0.7
40*	24	28	16	3.2	0.1	0.4
Analyzed diets						
IM	73 ± 3.7	71.7 ± 5.7	289 ± 18.8	9.82 ± 0.7	0.318 ± 0.021	–
AACM-100	77.3 ± 4.2	77.5 ± 3.9	307 ± 16.3	11.5 ± 0.6	0.329 ± 0.018	–
AACM-70	52.6 ± 2.7	54.2 ± 3.3	268 ± 15.8	7.15 ± 0.4	0.282 ± 0.015	–
AACM-40	39.8 ± 2	39.2 ± 2.1	239 ± 13.9	5.2 ± 0.3	0.24 ± 0.014	–
LGCM-100	74.2 ± 4	72.7 ± 4.2	296 ± 15.1	9.83 ± 0.6	0.317 ± 0.017	–
LGCM-70	53.9 ± 3	53.3 ± 3	268 ± 15.3	7.57 ± 0.4	0.259 ± 0.015	–
LGCM-40	41.1 ± 2.3	33 ± 1.9	216 ± 11	4.9 ± 0.3	0.22 ± 0.013	–
Water	0.175	1	0	0.025	0.085	

*AACM or LGCM. AACM, amino acid-complexed minerals; 100% (sole source), 70, and 40% of the total mineral requirements. LGCM, lysine and glutamic acid-complexed minerals; 100% (sole source), 70, and 40% of the total mineral requirements.

In Experiment 1, AACM premixes contained Zn, Mn, Cu, and Fe complexed with unspecified amino acids, while I was provided as Zn–I–AA complex, and Se was supplied as Zn–L–Se-Methionine. In Experiment 2, LGCM premixes contained Zn, Mn, Cu, Fe, and I complexed specifically with lysine and glutamic acid, while Se was provided as Zn–L–Se–Methionine. Both AACM and LGCM were supplied by Zinpro Performance Minerals^®^ (Eden Prairie, MN, United States). All amino acids provided per the mineral premixes are shown in Supplementary material.

The control diets contained an inorganic mineral (IM) premix formulated with Zn oxide (ZnO), Mn dioxide (MnO₂), Cu sulfate (CuSO₄), ferrous sulfate (FeSO₄), calcium iodate [Ca(IO₃)₂], and sodium selenite (Na₂SeO₃). All diets were manufactured at Federal Rural University of Pernambuco in a single batch per treatment to minimize variation. After mixing, representative feed samples were collected for subsequent analysis.

Dry matter content was determined by oven-drying at 105 °C for 24 h. The Kjeldahl method (*N* × 6.25) was used to quantify crude protein. Calcium and phosphorus levels were measured using spectrophotometry. For trace mineral concentrations, such as zinc, manganese, copper, iron, selenium, and iodine, atomic absorption spectrophotometry was employed, adhering to the protocols by Silva and Queiroz (21).

### 2.5 Eggshell cuticle quality analysis

Eggshell cuticle quality was assessed when hens were 89 weeks of age. Three eggs per replicate cage were randomly collected (*n* = 24 eggs per treatment) for analysis. Eggs were individually weighed on a digital scale (precision: 0.01 g) and labeled for identification. The cuticle was stained by immersing each egg in an aqueous solution containing 1% Cuticle Blue^®^ (MS Technologies Ltd., Northamptonshire, United Kingdom) for 5 min at room temperature (25 ± 2 °C). After staining, eggs were rinsed with distilled water to remove excess dye and air-dried on a wire rack at room temperature for 30 min.

Cuticle coverage was evaluated in three regions of each eggshell (basal, equatorial, and apical) using a spectrophotometer (Konica Minolta CR-410C, Konica Minolta Sensing Americas, Inc., Ramsey, NJ, United States) with a D65 illuminant and 2° standard observer. The spectrophotometer was calibrated using a standard white plate before measurements. Color parameters were calculated using the CIE L*a*b* color space system, where L* indicates lightness, a* represents the red-green component, and b* represents the yellow–blue component. The total color difference (*Δ*E*ab) was calculated using the formula:

$$\Delta E_{\mathrm{ab}}=\sqrt{{\left(\mathrm{\Delta L}\right)}^{2}+{\left(\mathrm{\Delta a}\right)}^{2}+{\left(\mathrm{\Delta b}\right)}^{2}}$$

where ΔL* is the difference between lighter and darker colors, Δa* is the difference between red and green, and Δb* is the difference between yellow and blue. A higher ΔE*ab value indicates greater color intensity and greater cuticle thickness (22).

### 2.6 Experimental egg contamination

For the bacterial penetration study, eggs were collected when hens were 86 weeks of age. A total of 182 eggs with an average weight of 64.9 ± 6.5 g were selected. Eggs with visible cracks or abnormalities were excluded. The eggs were randomly assigned to experimental treatments, with 56 eggs per treatment (in three replicates) plus 14 eggs that served as negative controls.

A nalidixic acid and rifampicin-resistant strain of *S.* Enteritidis phage type 4 was used for the experimental contamination. The bacterial strain was cultured in Brain Heart Infusion broth (BHI; Difco, Detroit, MI, United States) at 37 °C for 24 h. The culture was then centrifuged at 3,000 × g for 15 min at 4 °C, and the pellet was resuspended in buffered peptone water (BPW; Difco) to achieve a concentration of approximately 4.74 log₁₀ CFU/mL, as determined by spectrophotometry (Thermo Scientific^®^, model Genesis 10S UV–Vis). The actual concentration was confirmed by plate counting on Bright Green agar supplemented with nalidixic acid (100 μg/mL) and rifampicin (100 μg/mL).

Eggs were immersed for 3 min in the inoculum solution at room temperature (25 ± 2 °C). After inoculation, eggs were placed on sterile plastic-lined trays and stored at 26.3 ± 2 °C with a relative humidity of 62 ± 2% for 48, 96, 168, or 216 h. Temperature and humidity during storage were monitored using calibrated data loggers (HOBO U12-013).

### 2.7 Microbiological analysis

At each storage time point, bacterial counts were performed on both eggshell and yolk samples. For eggshell bacterial counts, each egg was placed in a sterile plastic bag containing 50 mL of 1% BPW and gently rubbed by hand for 1 min to detach bacteria. An aliquot (1 mL) of this rinsate was serially diluted (10-fold) in 9 mL of 1% BPW up to 10^−6^. From each dilution, 100 μL was spread-plated onto Bright Green agar supplemented with nalidixic acid (100 μg/mL) and rifampicin (100 μg/mL). The plates were incubated at 40 °C for 24 h, after which colonies were counted using a colony counter equipped with a magnifying glass and backlight.

For egg yolk bacterial counts, the same egg used for eggshell analysis was surface-disinfected by immersion in 70% ethanol for 3 min. After air-drying at room temperature in a biosafety cabinet, the egg was aseptically broken, and the yolk was separated from the albumen using a sterile yolk separator. Approximately 10 ± 2 g of yolk was aseptically transferred to a sterile bag containing 90 mL of 1% BPW and homogenized for 1 min in a stomach (400 Circulator, Seward, United Kingdom). Serial dilutions and plating were conducted as described for eggshell samples.

Colony counts were expressed as colony-forming units (CFU) and converted to log₁₀ CFU/mL for eggshell samples and log₁₀ CFU/g for yolk samples. The results were categorized into three risk levels for consumption: no risk (absence of *Salmonella*), moderate risk (log₁₀ ≤ 2.9), and high risk (log₁₀ ≥ 3.0) (23).

Negative control eggs were tested for the absence of *Salmonella* by enriching 25 g of eggshell or yolk in 225 mL of 1% BPW, followed by incubation at 40 °C for 24 h. Subsequently, 0.1 and 1 mL aliquots were transferred to Rappaport–Vassiliadis broth and Tetrathionate broth, respectively, and incubated at 40 °C for 24 h. A loopful from each broth was then streaked onto Brilliant Green Agar (BGA) and Xylose Lysine Deoxycholate Agar (XLD) and incubated at 40 °C for 24 h. Plates were examined for typical *Salmonella* colonies.

### 2.8 Verification of *Salmonella* Enteritidis penetration by scanning electron microscopy

**Figure 1:**
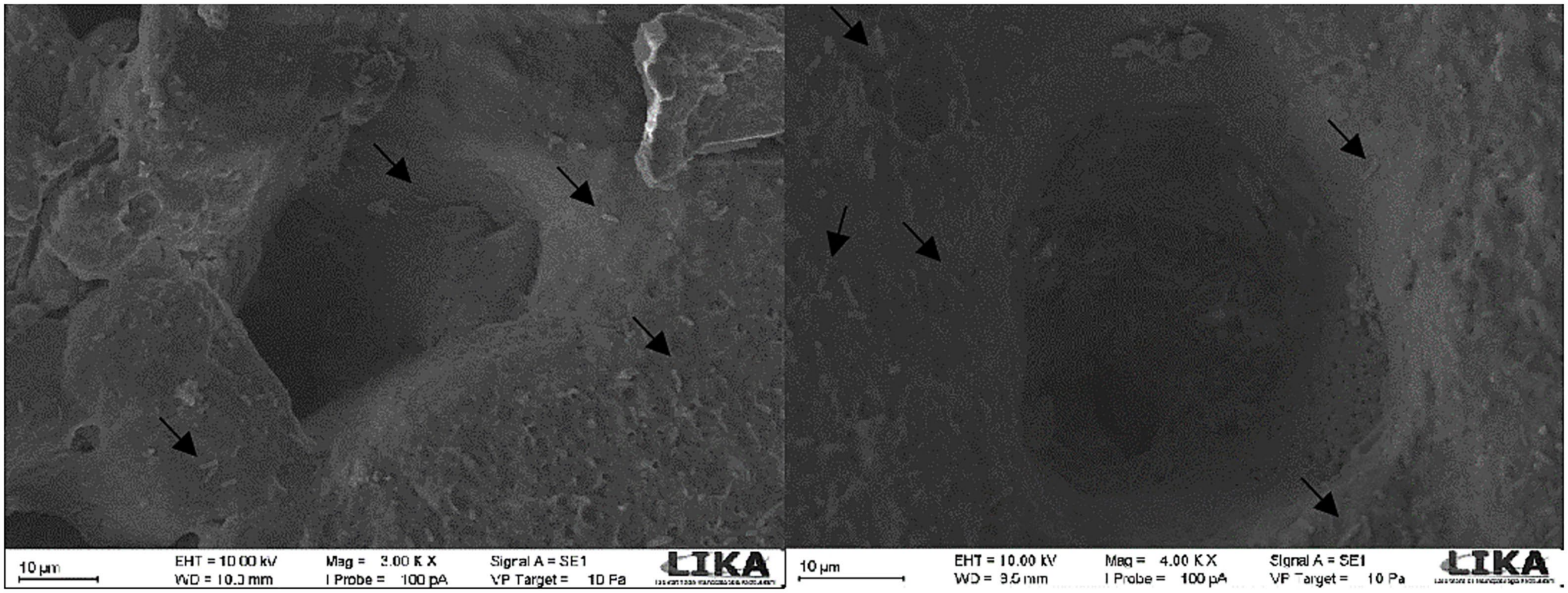
Visualization of *S.* Enteritidis in eggshell pores by scanning electron microscopy, indicated by arrows.

To visualize bacterial penetration through the eggshell, one egg per treatment at each storage time point was sampled for scanning electron microscopy (Figure 1). After experimental contamination and storage, a 1-cm^2^ fragment from the equatorial region of each eggshell was carefully removed using a micro grinder (Dremel Model 3,000, 120 W, 220 V) fitted with a cutting disk (23 mm diameter, 0.8 mm thickness).

The fragments were immediately fixed in Karnovsky solution (0.1 M phosphate buffer containing 2.5% glutaraldehyde and 4% formaldehyde, pH 7.2) and transported to the Keizu Asami Immunopathology Laboratory (LIKA/UFRPE). The samples were mounted on metallic stubs, dried in an oven at 35 °C for 10 min, and sputter-coated with gold using a metallizer (JFC-1100). The prepared specimens were examined using a scanning electron microscope (JOEL T-200) at 3,000 × magnification. Images were captured from at least three different areas per sample.

### 2.9 Statistical analysis

The experimental unit was the cage (*n* = 8 replicates per treatment), with five birds housed per cage. Sources of variation in the model included the fixed effect of dietary treatment and the random residual error associated with individual cage observations. Data were analyzed using the General Linear Model procedure in Statistical Analysis System software (version 9.2; SAS Institute Inc., Cary, NC, United States). Prior to analysis, data were tested for normality using the Shapiro–Wilk test and for homogeneity of variances using Levene’s test.

For eggshell cuticle quality data, the following statistical model was used:

$$Y_{\mathrm{ij}}=\mu +\mathrm{Ti}+e_{\mathrm{ij}}$$

where $Y_{\mathrm{ij}}$ is the observed value, μ is the overall mean, Ti is the fixed effect of treatment i, and $e_{\mathrm{ij}}$ is the random error. Data were analyzed using orthogonal contrasts to test linear and quadratic effects of inclusion levels within each mineral source. Additionally, Dunnett’s test at 5% probability was utilized to compare the IM treatments against AACM or LGCM levels. The *S.* Enteritidis penetration was analyzed using multiple unpaired *t*-tests using R-core software. Treatment effects were considered significant at *p <* 0.05.

## 3 Results

Experiment 1 evaluated amino acid-complexed minerals (AACM) at 100%, 70%, and 40% inclusion levels versus inorganic minerals (IM) in 67-week-old Dekalb White laying hens.

### 3.1 Eggshell quality

All negative control eggs tested negative for *Salmonella* Enteritidis, confirming the absence of pre-existing contamination. The Dunnett’s test revealed no significant differences between AACM treatments and the IM control for cuticle coloration (*p =* 0.33), mammillary knob width (*p =* 0.30), and mammillary layer (*p =* 0.40) (Table 3).

**Table 3:** Intensity of cuticle staining and shell layers of eggs obtained from laying hens supplemented with AACM.

Treatments^£^	Cuticle staining	Mammillary knob width (μm)	Mammillary layer (μm)	Palisade layer (μm)	Shell thickness (μm)
IM	46.1	101.0	87.0	224*	322*
AACM-100	45.4	87.3	78.1	244	350*
AACM-70	45.0	92.2	82.7	241	329
AACM-40	47.7	92.6	77.9	264*	345*
Mean	46.1	93.3	81.4	243	336
SEM	1.18	2.71	2.21	3.99	3.76
Dunnet-test	0.328	0.303	0.399	0.009	<0.001
Orthogonal contrasts					
Linear	0.122	0.451	0.982	0.126	0.487
Quadratic	0.142	0.717	0.354	0.183	0.003

IM, 100% Inorganic minerals; AACM, amino acid-complexed minerals; tested at levels of 100% (sole source), 70, and 40% of the total mineral requirements. *Differs from Dunnet test at 5% of significance. ^£^Experiment 1.

However, significant differences were observed for the palisade layer and shell thickness. The 40% AACM treatment exhibited higher palisade layer values than IM (*p <* 0.01), and both 100% AACM and 40% AACM showed increased shell thickness compared to the IM control (*p <* 0.01).

The polynomial orthogonal contrast indicated that treatments did not influence cuticle staining (*p =* 0.14), mammillary knob width (*p =* 0.72), mammillary layer (*p =* 0.35), or palisade layer (*p =* 0.18). Shell thickness demonstrated a quadratic trend to AACM inclusion (*p <* 0.01).

### 3.2 *Salmonella* Enteritidis contamination

When evaluating *S.* Enteritidis recovery from eggshells and yolks over time (48–216 h post-inoculation), no consistent contamination pattern was observed between control and AACM treatments (Table 4). The eggshell contamination levels remained similar among all treatments. However, egg yolks from laying hens supplemented with 70 and 40% AACM showed reduced (3.59 lower) *S.* Enteritidis counts despite similar external eggshell contamination.

**Table 4:** Recovery of *Salmonella* Enteritidis in eggshell and yolk over time and average counts of *S.* Enteritidis in eggshell and yolk in eggs obtained from laying hens supplemented with AACM.

Treatments^£^	100% IM	100% AACM	70% AACM	40% AACM
Recovery ( $\mathrm{lo}g_{10}$ ) of *S.* Enteritidis from eggshell over time				
48 h	2.43	4.18	4.27	4.70
96 h	3.20	3.09	5.78	5.03
168 h	2.91	2.87	4.13	3.38
216 h	2.75	5.06	4.74	2.98
Recovery (log₁₀) of *S.* Enteritidis from yolk over time				
48 h	0	0	5.78	1.72
96 h	0	0	0	0
168 h	4.55	2.76	1.01	0
216 h	0	2.44	3.33	0

IM, 100% inorganic minerals; AACM, amino acid-complexed minerals; tested at levels of 100% (Sole Source), 70, and 40% of the total mineral requirements. ^£^Experiment 1.

**Figure 2:**
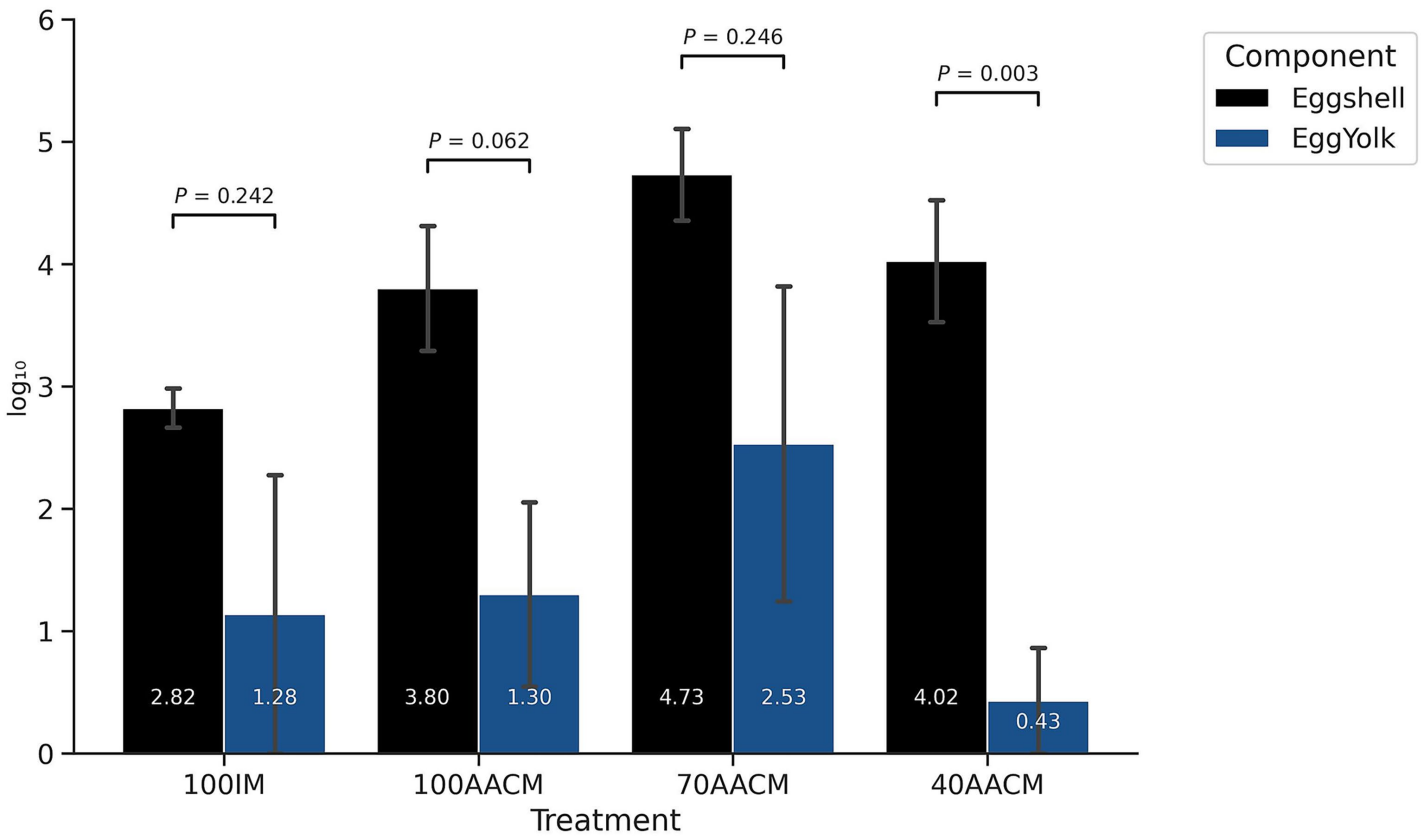
Mean recovery of *S.* Enteritidis in eggshell and yolk obtained from laying hens supplemented with AACM. IM, 100% inorganic minerals; AACM, amino acid complexed-minerals; tested at levels of 100% (Sole Source), 70, and 40% of the total mineral requirements. Error bars represent the standard deviation (SD).

**Figure 3:**
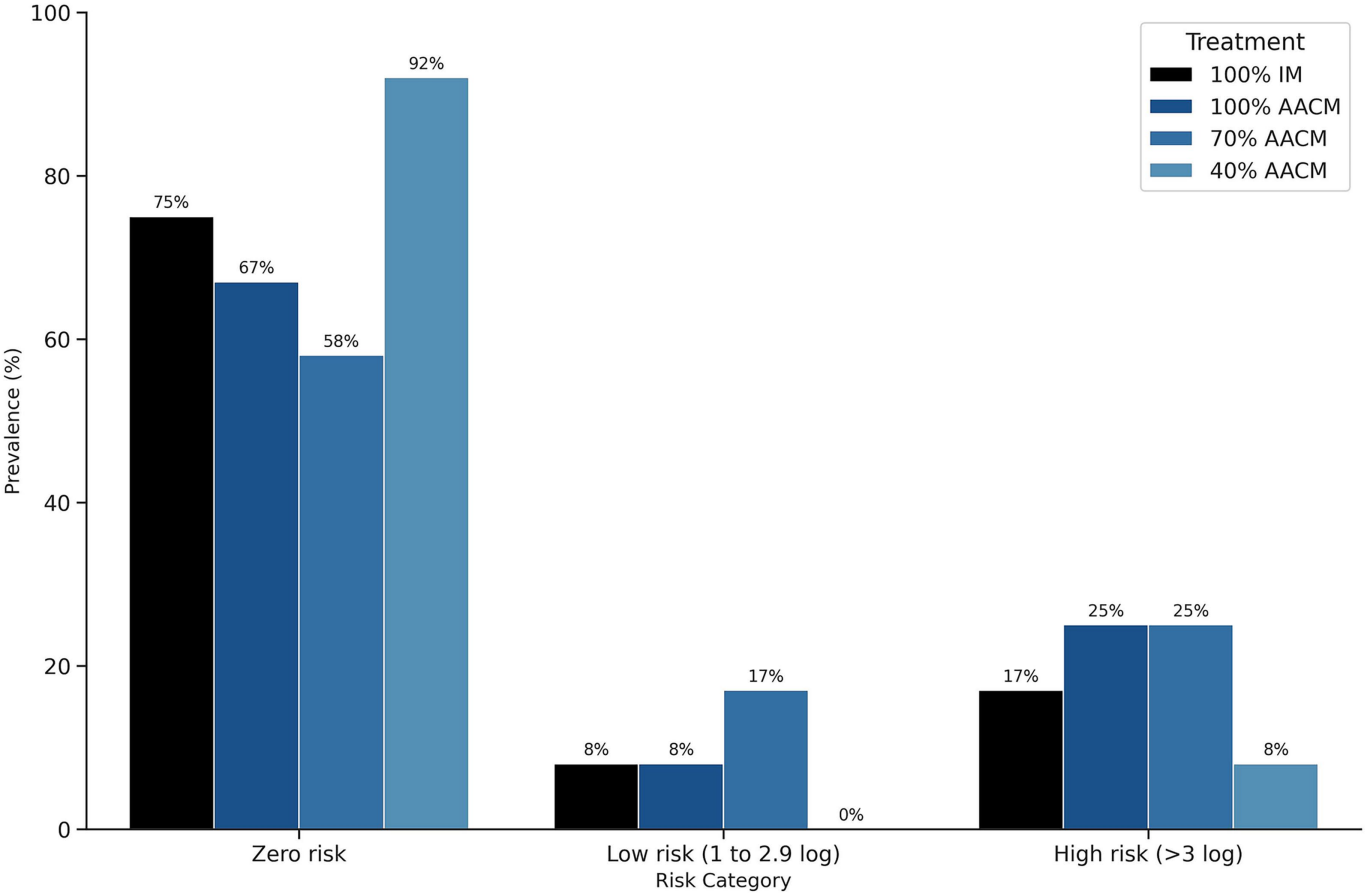
Prevalence of *S.* Enteritidis in yolks of eggs obtained from laying hens supplemented with AACM. IM, 100% inorganic minerals; AACM, amino acid complexed-minerals; tested at levels of 100% (sole source), 70, and 40% of the total mineral requirements.

There was no statistical difference in counts of *S.* Enteritidis in yolks versus eggshell in the IM control, and 100 and 70% of AACM (Figure 2). However, in the 40% AACM treatment, *S.* Enteritidis counts in egg yolks were significantly lower than those in eggshells (*p <* 0.01). The risk classification (Figure 3) showed that 40% AACM treatment yielded the highest percentage of samples classified as no risk, while IM, 100% AACM, and 70% AACM were associated with a greater proportion of samples in the moderate to high-risk categories.

Experiment 2 assessed lysine and glutamic acid-complexed minerals (LGCM) at the same inclusion levels (100%, 70%, and 40%) versus inorganic minerals (IM) in 67-week-old Dekalb White laying hens.

### 3.3 Eggshell quality

The Dunnett’s test showed no significant differences between LGCM treatments and the IM control for mammillary knob width (*p =* 0.11) and shell thickness (*p =* 0.15) (Table 5). Both 70% LGCM and 40% LGCM exhibited higher cuticle staining values than IM (*p <* 0.01). The mammillary layer was significantly lower in 100% LGCM compared to IM (*p =* 0.01), and the palisade layer was significantly thicker in 100% LGCM relative to the control (*p <* 0.01).

**Table 5:** Intensity of cuticle staining and shell layers of eggs obtained from laying hens supplemented with LGCM.

Treatment^¥^	Cuticle staining	Mammillary knob width (μm)	Mammillary layer (μm)	Palisade layer (μm)	Shell thickness (μm)
IM	46.1*	105	97.5*	224*	322
LGCM-100	48.0	83.0	76.0*	255*	329
LGCM-70	51.1*	90.6	85.9	225	313
LGCM-40	49.6*	97.4	85.6	240	330
Mean	48.7	94.0	86.3	236	324
SEM	1.88	2.68	2.33	4.31	2.62
Dunnet	<0.001	0.105	0.014	0.005	0.149
Orthogonal contrasts					
Linear	0.068	0.002	0.025	0.104	0.94
Quadratic	<0.001	0.901	0.172	0.006	0.035

IM, 100% inorganic minerals; ^¥^Experiment 2. LGCM, lysine and glutamic acid-complexed minerals; evaluated at levels of 100% (Sole Source), 70, and 40% of the total mineral supplementation. *Differs from Dunnet test at 5% significance.

Linear trends for mammillary knob width (*p <* 0.01) and mammillary layer (*p =* 0.03) were observed in the LGCM treatments, while quadratic trends were observed for cuticle staining (*p <* 0.01), palisade layer (*p <* 0.01), and shell thickness (*p =* 0.04).

### 3.4 *Salmonella* Enteritidis contamination

After 48 h of experimental contamination, the 40% LGCM treatment showed lower recovery of *S.* Enteritidis from eggshells compared to the IM control group (Table 6). Considering average values over time, the lowest recovery in egg yolks was obtained from birds fed with 100% LGCM. After 216 h of shell contamination, no recovery of *S.* Enteritidis was observed in egg yolks from any treatment.

**Table 6:** Recovery of *S.* Enteritidis in eggshell and yolk over time and average counts of *S.* Enteritidis in eggshell and yolk in eggs obtained from laying hens supplemented with LGCM.

Treatments^¥^	Recovery ( $\mathrm{lo}g_{10}$ ) of *S.* Enteritidis from eggshell over time			
	100% IM	100% LGCM	70% LGCM	40% LGCM
Recovery (log10) of *S.* Enteritidis from eggshell over time				
48	2.43	3.94	4.84	5.32
96	3.20	2.59	3.39	4.37
168	2.91	4.00	2.71	2.87
216	2.75	3.15	4.25	1.56
Recovery ( $\log_{10}$ ) of *S.* Enteritidis from yolk over time				
48	0	0	0	0
96	0.57	0	0	0
168	4.55	0	0	0
216	0	0	0	0

IM, 100% inorganic minerals; LGCM, lysine and glutamic acid-complexed minerals; evaluated at levels of 100% (sole source), 70, and 40% of the total mineral supplementation. ^¥^Experiment 2.

**Figure 4:**
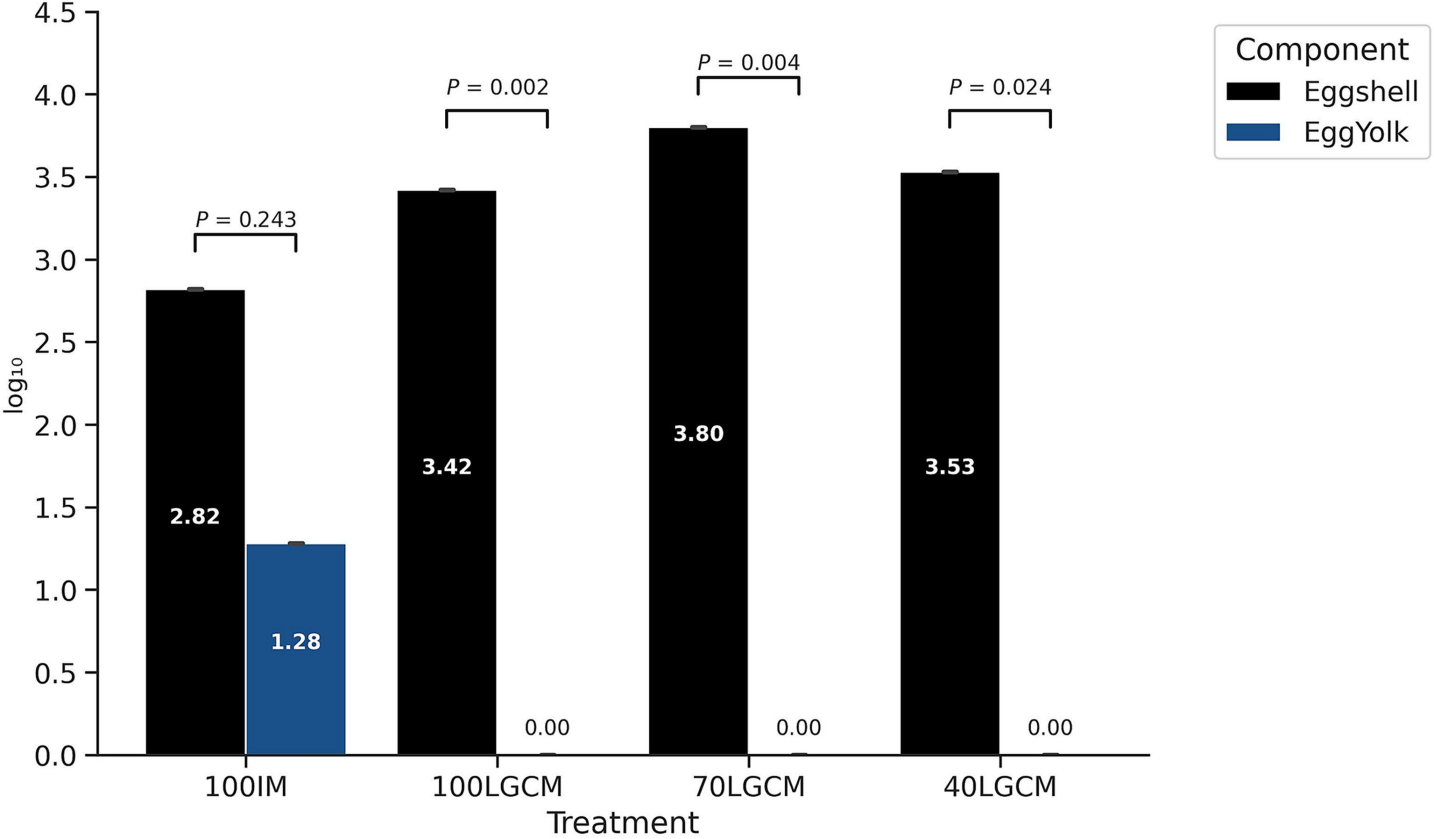
Mean recovery of *S.* Enteritidis in eggshell and yolk obtained from laying hens supplemented with LGCM. IM, inorganic minerals; LGCM, lysine and glutamic acid-complexed minerals, evaluated at levels of 100% (sole source), 70, and 40% of the total mineral supplementation. Error bars represent the standard deviation (SD).

**Figure 5:**
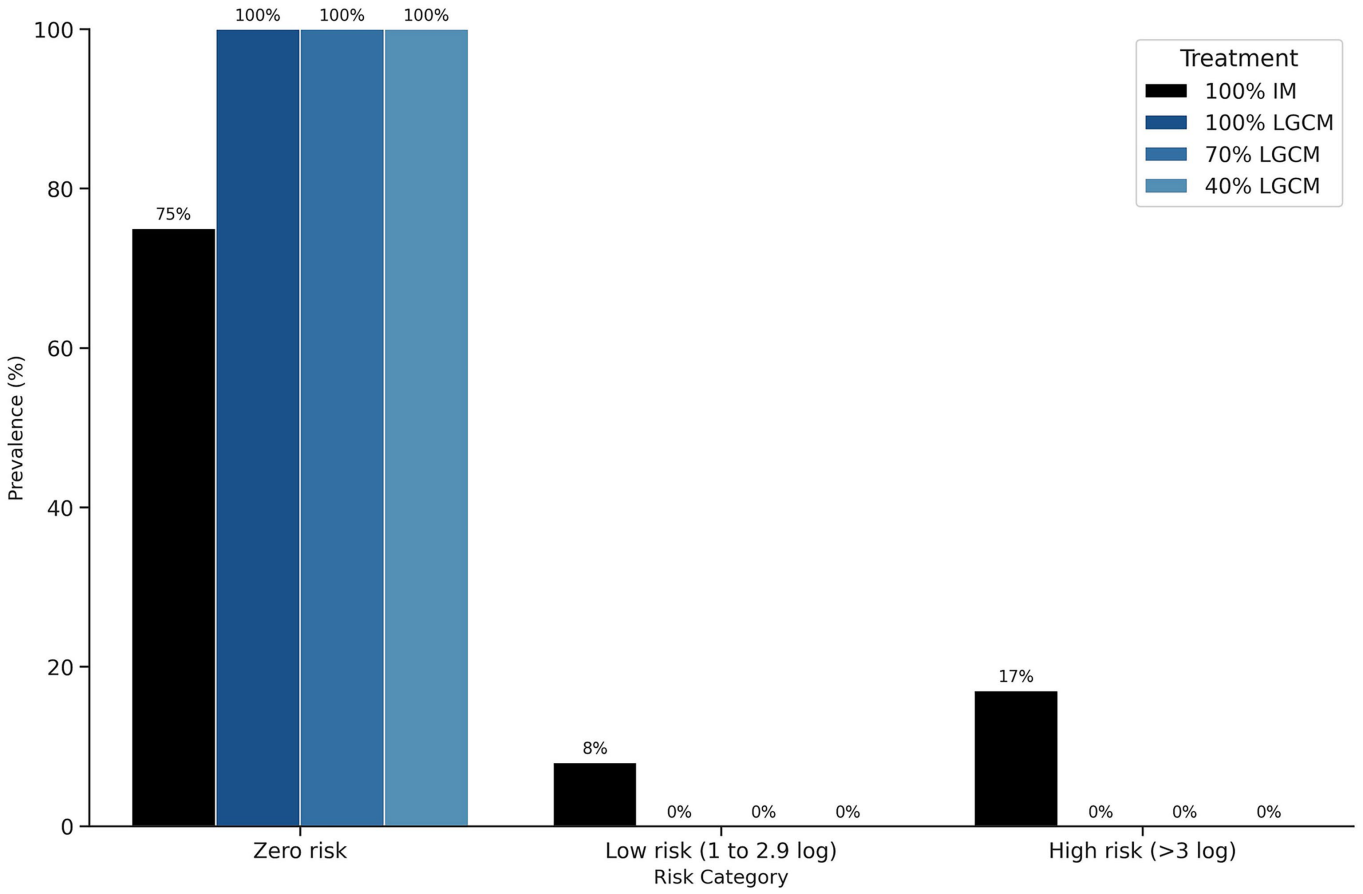
Prevalence of *S.* Enteritidis in yolks of eggs obtained from laying hens supplemented with LGCM. IM, inorganic minerals; LGCM, lysine and glutamic acid-complexed minerals; evaluated at levels of 100% (Sole Source), 70, and 40% of the total mineral supplementation.

Significant differences in *S.* Enteritidis recovery between eggshells and yolks were observed across all LGCM treatments (*p <* 0.01), with egg yolks exhibiting zero values (Figure 4). In contrast, the IM treatment did not show a significant difference between eggshell and yolk contamination levels (*p =* 0.24). As presented in Figure 5, LGCM treatments achieved 100% of samples with no risk, whereas the IM treatment had 8–17% of samples categorized as low to high risk.

## 4 Discussion

The experiments revealed interesting, time-dependent patterns of *S.* Enteritidis contamination on eggshells. Although initial observations at 48 h post-contamination showed higher *Salmonella* recovery on eggshells from hens fed 100% IM and 70% AACM diets, this trend did not persist after 216 h. Subsequent samples exhibited significant fluctuations in *Salmonella* counts across different treatments and time points, suggesting that initial observations might not fully capture the impact of dietary treatments on eggshell susceptibility to *Salmonella* contamination. A more comprehensive analysis, considering the average *Salmonella* recovery across all time points, provides a more robust evaluation of the observed effects. This variability highlights the importance of bacterial load on the eggshell surface in determining bacterial penetration into egg contents. Research by Howard et al. (24) demonstrated that *S.* Enteritidis penetration depends on factors such as temperature differential, humidity, microbial concentration, and storage conditions, all of which were controlled in this study, indicating that the observed differences are attributable to dietary treatments. The penetration of *Salmonella* spp. from the eggshell surface to the internal contents can be initiated within the first 24 h post-contamination. However, the complete bacterial migration to the yolk is a more extended process, and its kinetics are modulated by multiple factors. These include the storage temperature, which accelerates translocation under non-refrigerated conditions, the physicochemical properties of the shell, such as its porosity and humidity, and the integrity of the vitelline membrane, which functions as a crucial biological barrier. Therefore, establishing a 216-h analysis period in the present study was a methodological decision to ensure sufficient time for the detection of potential bacterial penetration, even in slow-migration scenarios, such as those observed in eggs kept under refrigeration (25). Messens et al. (26) examined the survival and penetration of *Salmonella enterica* serovar Enteritidis on eggshells stored for up to 20 days under real-life conditions (15–25 °C and 45–75% relative humidity). They found that while the number of surviving organisms decreased over time, *Salmonella* could still be recovered.

The 40% AACM produced the most pronounced increase in both palisade layer and shell thickness and resulted in the lowest *S.* Enteritidis counts in yolk. Therefore, the improved shell integrity may have contributed to decreased bacterial translocation from the shell to the yolk (27–29).

In Experiment 2, a notable difference was observed when hens were supplemented with 70% LGCM, contributing to enhanced cuticle coverage. Remarkably, at all inclusion levels of LGCM, *S.* Enteritidis was not detected in egg yolks. These results align with previous studies demonstrating that thicker cuticle deposition effectively reduces bacterial penetration into eggs (30–32). The cuticle serves as a natural defense against microbial penetration through the eggshell, with thicker cuticles being associated with lower rates of bacterial penetration (31, 33). Despite higher *S.* Enteritidis counts on eggshells from birds supplemented with LGCM, no contamination was observed in the yolks. This can be attributed to improved cuticle coverage that prevented *Salmonella* penetration. Minerals complexed with organic molecules like amino acids have higher absorption rates because their uptake is mediated by amino acid transporters, reducing competition for absorption at binding sites (19). Pereira et al. (34) found that laying hens fed diets containing AACM had longer intestines, which likely increased the nutrient absorption area. In another experiment with AACM supplementation, Medeiros–Ventura et al. (17) reported reduced phosphorus excretion in Lohmann Brown birds from 1 to 35 days of age, suggesting improved dietary utilization and consequently enhanced eggshell quality in the egg output phase.

Egg quality is also determined by eggshell thickness (16). Several studies indicate that simultaneous supplementation with organic amino acid complexes of Zn, Mn, and Cu increases eggshell thickness (35, 36). Sauer et al. (37) and Gao et al. (19) reported that minerals complexed with amino acids can utilize less saturable pathways for absorption. Their *in vitro* studies with Caco-2 cells have shown that metals complexed to amino acids utilize their respective transporters, increasing uptake on the apical side or efflux on the basolateral side. The improved uptake and effective absorption of amino acid-complexed minerals partially explain the findings in our study.

Each trace mineral plays an important role in eggshell formation and egg protection. The Zn acts as a cofactor for carbonic anhydrase, an enzyme involved in eggshell formation (38). Shao et al. (39), supplementing Zn in broiler diets challenged with *S.* Typhimurium, reported that Zn repaired intestinal damage, increased villous height and ileal epithelial cell proliferation, and regulated the cecal microbial community. Anderson et al. (40) demonstrated that zinc–amino acid and zinc plus manganese–amino acid complexes significantly reduced *Salmonella* Typhimurium in broilers. Birds fed these complexes showed lower fecal shedding and cecal colonization of *Salmonella* compared to control groups.

As a component of AACM and LGCM, Mn can modulate eggshell quality by improving the activity of Gal β1,3-glucuronosyltransferase (GLcAT-I), which participates in proteoglycan synthesis (41). The Mn supplementation can enhance the expression of genes encoding proteoglycans and glycoproteins in the eggshell gland, resulting in increased mammillary button density during the initial shell formation stage (42). Additionally, Mn contributes to eggshell quality by participating in mucopolysaccharide synthesis, which is important for eggshell organic matrix formation (13). The organic matrix conformation is crucial for eggshell quality as it determines how and when nucleation sites will be deposited, upon which the eggshell crystalline structure develops. Cui et al. (43) evaluated the effects of amino acid-complexed Mn supplementation and reported improvements in productive performance and eggshell breaking strength.

Activation of lysyl oxidase by Cu is necessary for collagen synthesis in the eggshell membrane (44). This enzyme catalyzes the oxidative deamination of lysine side chains, forming cross-links that confer insolubility, flexibility, and structural characteristics for the deposition of other eggshell components (45, 46). The higher bioavailability of I complexed minerals may contribute to the reduction in *S.* Enteritidis penetration. Iodine has relevant antibacterial functions in egg yolk when incorporated into the diet (47, 48). Damaziak et al. (49) found that dietary I supplementation in commercial laying hens effectively inhibited *Salmonella enterica* growth in eggs incubated at 30 °C for up to 10 days, and high I levels strongly inhibited *S. enterica* migration from egg white to yolk.

In Experiment 2, laying hens supplemented with LGCM showed superior results, as *S.* Enteritidis could not penetrate and contaminate egg contents in any treatment at any inclusion level. Generally, the results obtained with AACM and LGCM were superior to those obtained with IM. This is because IMs dissociate into active cations for absorption when they reach the gastrointestinal tract, potentially causing mineral interactions and competition at enterocyte absorption sites. Laying hens actively require Ca to form amorphous calcium carbonate (50) and calcium phosphate during eggshell calcification (51). However, before Ca and P are utilized for bone and eggshell formation, trace minerals, primarily Zn, Mn, and Cu, play fundamental roles in establishing the ultrastructure of these tissues, which forms the foundation for crystal structure development. Reducing dietary Fe content to even lower levels (70 and 40%) may further restrict bacterial access to this essential element, negatively impacting the growth and diversity of gut microbiota, such as *Salmonella* species. Another factor to consider is the competitive exclusion or predation of *Salmonella* by resident eggshell bacteria. All microbial growth eventually enters a decline phase (cell death), which depends on extrinsic factors such as humidity and temperature and intrinsic factors, such as substrate availability, water activity, acidity, oxygen, and chemical composition (52).

According to the Food and Agriculture Organization (23), the risk characterization of *S.* Enteritidis in eggs predicts that the probability of causing disease given an average dose of 1, 10, or 100 organisms is 0.2, 2.2%, or 13%, respectively. Regarding the risk for human consumption established in this study, in Experiment 1, the 40% AACM treatments showed the best results, with 92% of samples classified as having no risk for consumption. Moreover, the IM treatment resulted in 75% of its samples being classified as a risk for consumption. In Experiment 2, all LGCM treatments produced samples with no risk for consumption. These results have significant public health implications, considering the increasing global egg consumption from 15 to 90 million tons between 1961 and 2019 (53).

Controlling *S.* Enteritidis in eggs intended for human consumption is a constant challenge for the food industry. Our results demonstrate that supplementing laying hen diets with LGCM at any level prevented *S.* Enteritidis penetration, while 40% AACM reduced egg yolk contamination. However, further studies with laying hens of different ages and with specific trace mineral sources are necessary to better understand the responses of laying hens to AACM and LGCM supplementation in relation to egg quality and food safety.

The interaction between trace minerals and the gut microbiome justifies further consideration in this context. Research by Dong et al. (54) has shown that organic trace minerals can positively modulate the intestinal microbiota composition in laying hens, potentially creating a more competitive environment against pathogenic bacteria like *Salmonella*. A study by De Grande et al. (55) demonstrated that supplementing with Zn-amino acid complexes modulates intestinal morphology by increasing villus height and reducing crypt depth, while simultaneously altering cecal microbiota composition through decreased Firmicutes abundance and increased Bacteroidetes populations. This modulation of gut microbiota may contribute to the overall health of laying hens and, consequently, improve eggshell quality and reduce susceptibility to bacterial contamination.

Additionally, the economic implications of using amino acid-complexed minerals in poultry diets should be considered. While complex mineral sources typically cost more than inorganic alternatives, their enhanced bioavailability allows for reduced inclusion levels while maintaining or improving performance (16).

## 5 Conclusion

Experiment 1 demonstrated that 40% AACM supplementation in laying hen diets significantly improved eggshell quality by increasing palisade layer thickness and overall shell thickness. This treatment also provided the best protection against *S.* Enteritidis penetration, with 91.67% of egg samples classified as having no risk for consumption. Experiment 2 showed that LGCM supplementation at all inclusion levels prevented *S.* Enteritidis penetration into egg yolks. The 70% LGCM treatment particularly enhanced cuticle coverage, resulting in 100% of samples classified as having no risk for consumption despite eggshell contamination.

## Data Availability

The raw data supporting the conclusions of this article will be made available by the authors, without undue reservation.
