# Supplementary material for: The impact of metal amino acid complexes on cuticle quality and Salmonella Enteritidis contamination in laying hens’ eggs
Source: Front Vet Sci. 2026 Jan 30;12:1692361. doi: 10.3389/fvets.2025.1692361 (PMC12903117; doi:10.3389/fvets.2025.1692361)
Supplement: Supplementary file 1 [file Table_1.docx]

Supplementry Table 1. Amino acid content (% of the feed) of Cu, Mn, Zn and Fe complexed to amino acids

| **Amino Acid** | **Availa - Zn** | **Availa - Mn** | **Availa - Cu** | **Availa - Fe** |
| --- | --- | --- | --- | --- |
| Alanine, % | 0.400 | 0.995 | 0.009 | 0.22 |
| Arginine, % | 0.350 | 0.919 | 0.008 | 0.20 |
| Aspartic Acid, % | 0.425 | 1.072 | 0.010 | 0.24 |
| Glutamic Acid, % | 0.850 | 2.220 | 0.019 | 0.48 |
| Glycine, % | 0.475 | 1.225 | 0.011 | 0.27 |
| Histidine, % | 0.050 | 0.153 | 0.001 | 0.03 |
| Isoleucine, % | 0.300 | 0.766 | 0.007 | 0.17 |
| Leucine, % | 0.575 | 1.455 | 0.013 | 0.32 |
| Lysine, % | 0.150 | 0.383 | 0.003 | 0.08 |
| Methionine, % | 0.050 | 0.153 | 0.001 | 0.03 |
| Phenylalanine, % | 0.325 | 0.842 | 0.008 | 0.18 |
| Proline, % | 0.675 | 1.761 | 0.015 | 0.38 |
| Serine, % | 0.650 | 1.684 | 0.015 | 0.36 |
| Theonine, % | 0.275 | 0.689 | 0.006 | 0.16 |
| Tyrosine, % | 0.200 | 0.536 | 0.004 | 0.11 |
| Valine, % | 0.425 | 1.072 | 0.010 | 0.24 |
| Crude protein, % | 7.00 | 3.09 | 0.141 | 2.98 |

Amino acid content (% of the feed) of Cu, Mn, Zn and Fe complexed to lysine and glutamic acid

| **Amino Acid** | **LGCM - Zn** | **LGCM - Mn** | **LGCM - Cu** | **LGCM - Fe** |
| --- | --- | --- | --- | --- |
| Glutamic Acid, % | 0.243 | 0.412 | 0.004 | 0.333 |
| Lysine, % | 0.222 | 0.412 | 0.004 | 0.326 |
| Crude protein, % | 0.464 | 0.824 | 0.007 | 0.659 |
